# Ritual in Therapy for Prolonged Grief: A Scoping Review of Ritual Elements in Evidence-Informed Grief Interventions

**DOI:** 10.3389/fpsyt.2020.623835

**Published:** 2021-02-03

**Authors:** Joanna Wojtkowiak, Jonna Lind, Geert E. Smid

**Affiliations:** ^1^Department of Humanist Chaplaincy Studies, University of Humanistic Studies, Utrecht, Netherlands; ^2^ARQ National Psychotrauma Center, Diemen, Netherlands

**Keywords:** prolonged grief, traumatic grief, ritual intervention, therapy, treatment, evidence-based, evidence-informed

## Abstract

The aim of this article of to analyze ritual in evidence-informed treatments for prolonged and traumatic grief. A scoping review is conducted in order to give an overview of existing literature on ritual and symbolic interventions in grief therapies for prolonged grief and the type of evidence supporting these interventions. The 22 studies reported in this review reveal a variety of ritual elements ranging from symbolic expression and interaction, writing assignments, dialogue with the deceased or an imaginary person, to farewell ceremonies at the end of the treatment. The interventions are studied within different populations (e.g., bereaved spouses, perinatal loss, grief after violent death, and genocide). Almost all studies show significant effects of the grief treatment, trauma and related symptoms. However, the effects are mostly measured for the entire treatment and not separately for the ritual intervention. In the discussion we focus on the role of ritual and culture in prolonged grief treatment.

## Introduction

Grief comprises emotional pain related to the death of a loved one, feelings of yearning and longing, and preoccupation with the deceased person or the circumstances of the death (1, 2). The emotional pain can take the form of intense sadness or pangs of grief, bitterness, anger, guilt, denial, reproach, difficulty accepting death, a feeling of having lost part of oneself, feeling that life is meaningless, and difficulty in engaging with social or other activities. If these reactions cause clinically significant distress or impairments in functioning during more than 6–12 months, and if these reactions go beyond cultural norms for bereavement reactions and are not better explained by culturally specific mourning rituals, a diagnosis of a grief disorder may apply (1, 2). In this paper, we will use “prolonged grief” to denote symptoms associated with grief disorders that were assessed within the context of grief therapy. The death of a loved one due to violent causes can also cause post-traumatic stress disorder (PTSD), characterized by intrusive memories, attempts to avoid these, negative thoughts and mood and increased arousal, and reactivity. The term “traumatic grief” will be used in this paper to denote symptoms of prolonged grief and PTSD following the loss of a loved one due to violent or non-natural causes of death.

Rituals around grief and death have been practiced since the early beginnings of culture (3–5). Across countries, communities and historical periods, rituals have been a crucial element in dealing with grief and loss (6–9). Although many traditionally religious practices have been decreasing in Western societies, grief rituals remain in secular contexts (9). Rituals, as cultural enactments of meaning making, are still an important part of death culture across the world. Death rituals, such as funerals and memorials, are argued to help mourners transition into their new inner states and social statuses, such as from “wife” to “widow,” and to symbolically keep a bond with the deceased (10).

In the therapeutic literature, there has been increasing attention toward new types of treatment and interventions that include some kind of symbolic or ritual communication (11–13), such as a ritual of remembrance or renewal at the end of a treatment (14). These types of ritual acts in therapy are interesting, because they make use of elements from traditional rituals. The therapeutic context, where rituals are used, however, differs from traditional ritual, as it is used as intervention for coping with prolonged and traumatic grief and are argued to provide an important part to the grieving process, especially in prolonged or traumatic grief (12, 15). From an interview study with 10 psychotherapists, three types of grief ritual interventions were categorized: first, rituals to honor the deceased loved one and to keep a symbolic bond (12), second, rituals to let go of the traumatic experience, and third, rituals to self-transform (12). The interviews reveal that by using symbolic objects and acts, clients are able to enact their feelings into ritual action. Although the loss cannot be changed, rituals create an opportunity to express and enact painful emotions.

But what defines rituals in grief interventions? First, we need to establish what is understood as ritual more generally. In the ritual studies literature, there are at least 60 different scholar definitions of rituals (16). A starting point here are the four most common characteristics of rituals across cultures and contexts: rituals are an “*embodied, condensed* and *prescribed enactment”* [(16), p. 196]. This means that rituals are: (1) embodied action (not merely mental activity); (2) follow a certain protocol or structure; (3) differ from the ordinary or everyday use of language, action and objects and (4) need to be performed or enacted in a specific way.

Legare and Souza (17) argue that rituals “pose a cognitive paradox: although widely used to treat problems, they are cultural conventions and lack a causal explanation for their effects” [(17), p. 1], which additionally becomes more complicated by the fact that “as in few other human activities, the actors both are, and are not, the author of their acts” [(18), p. 5]. People actively conduct rituals and simultaneously are “conducted” by the ritual.

Rituals are also defined in terms of “a lack of instrumental purpose” [(19), p. 73; (20), p. 2] and “goal-demotion” [(21), p. 598; (17), p. 2]. The notion of ritual efficacy is still debated (17, 22). Rituals have been defined as “causally opaque” [(17), p. 1], while also regulating emotions, performance and social connection (19, 20). Rituals thus might have a purpose, perhaps not an obvious, explicit or rational one. The ritual a whole might serve a purpose, the subsequences, subparts or singular acts of the ritual, are not necessarily experienced in such a way, possibly due to the cognitive overload, and emotional arousal during ritual (21). As rituals strongly focus on emotional, sensory, embodied and attentive processes, they do serve the need to invest time into a specific, prescribed activity. From a cognitive perspective, this means that the intention to invest time in a “causally opaque” activity, is motivational, and therefore already “serves a purpose.”

Although symbols and symbolic communication are a central aspect of rituals, ritual experiences are not merely a symbolic expression of a certain belief or attitude (22, 23). Instead, rituals create a symbolic, alternative reality that enables meaningful actions. This means that within a ritual, participants psychologically experience the ritual as “real” or authentic [(11), p. 40]; they feel in control while at the same time, they become one with the ritual, which at the same time gives a sense of letting go. Goodwyn (11) writes that participants know that there is a symbolic connection between the act, object and the meaning, without feeling that what they are doing is merely “symbolic” (p. 40). The ritual creates a safe environment to express emotions as the ritual has a clear beginning and end. Furthermore, the ritual is an aesthetic translation of reality through the use of specific attributes, such as candles or flowers, which creates an emotionally safe space to feel painful emotions (24). The emotions felt during ritual are authentic, but the context is “artificial” in the sense of being culturally created (16).

In some intense rituals, participants feel that they become one with the ritual, which happens when the boundaries of one's self become vague and one feels unified with the ritual (25). This is what Rappaport (26) describes as “high-order meaning” (p. 73) and Whitehouse and Lanman (25) define as “identity fusion” (p. 676). Becoming one with the ritual experience means that the meanings given are grounded in one's identity and deepest inner self. The ritual offers symbols to be able to link one's memories and identity with the ritual. Whitehouse and Lanman (25) argue that in identity fusion aspects of one's episodic memory are linked to the experience, in contrast to group identification when one feels that some prototypical features are shared (p. 676). In ritual, the symbolic, imagined world and the real world become one (27). This unique and specific characteristic might be an important reason why rituals have been integrated in grief therapy (15). When grief is particularly traumatic or problematic, the bereaved remain in a “persistent ambivalent state in which the bereaved simultaneously refuses to accept the loss of the loved one while also recognizing the stark reality of loss” [(28), p. 240]. Ritual creates a possibility to act on that ambivalence and through the use of symbolic language and acts the ambivalence in not denied or rejected, instead it is embraced. Liénard and Boyer (29) focus on the concept of “ritualized behavior,” instead of the general category of ritual, to stress the notion of “a specific way of organizing the flow of behavior, characterized by compulsion (one must perform the particular sequence), rigidity (it must be performed the right way), redundancy (the same actions are often repeated inside the ritual) and goal demotion (the actions are divorced from from their usual goals)” (p. 815). For the sake of clarity in this study, we define rituals in therapeutic interventions as*, sensory, attentive and intentional acts that are performed in a structured, imaginative or aesthetic way and make use of symbols, symbolic language, and symbolic action*.

While there is growing interest in ritual in grief therapy, there has not been a systematic overview of ritual use in evidence-based and evidence-informed therapy yet. By evidence-informed we refer to interventions that can be scored on the levels of evidence as developed by the Oxford Center for Evidence-Based Medicine (30). The aim of this article is to systematically investigate what kind of rituals and ritualized acts are used in grief interventions for prolonged grief and what kind of evidence is described for their effectiveness in terms of reducing symptoms of prolonged grief and/or PTSD following the loss of loved ones.

The research question is: *what kind of ritual elements are found in evidence-informed grief interventions for prolonged and traumatic grief?* By ritual elements we refer to specific acts within the interventions that include ritualized or symbolic enactments that are related to the grief experience. The research question will be approached by a scoping review (31). A scoping review is a synthesis of the literature that incorporates different search strategies (32). By conducting this scoping review on ritual in grief therapy, we will analyze what kind of rituals elements are used in evidence-informed grief therapy and what kind of evidence there is for the use of ritual in therapy. The overall aim of this study is to add knowledge on the importance of cultural processes in grief therapy. From studying existing literature on grief interventions with ritual elements we can learn what kind of ritual acts do help in dealing with prolonged grief.

## Methods

### Search Strategy

In order to answer the research question, we conducted a scoping review (31) through an online bibliographic search of databases and search systems. We used the key publications in Table 1 to give input into our search strategy. More conventional bibliographic databases and databases of guidelines, (ongoing) trials and gray literature were included. We searched the following online search systems: PsycINFO (Ovid), Ovid Medline, Embase (Ovid), Ovid Evidence Based Medicine Reviews, PTSDpubs, TRIP database, The National Institute for Health and Care Excellence (NICE), International Clinical Trials Registry Platform (ICTRP), and OpenGrey.

**Table 1:** Evidence-informed psychotherapies for traumatic grief comprising symbolic interactions with the deceased person.

**Name**	**Reference**	**Interaction**	**Description**
Complicated Grief Treatment	(33)	Dialogue	Imaginary conversation with the deceased
Integrated Cognitive Behavioral Therapy for Grief	(34)	Dialogue	Walk to the grave: what I always still wanted to tell and ask, how your death has impacted my life
		Ritual	Dedicate a memento
Writing Therapy for Grief	(35)	Letter	Letter to imagined significant other
Finding Meaning in Loss	(14)	Letter	Hello again letter, letter from loved one
		Ritual	Ritual of remembrance or a ritual of renewal
Brief Eclectic Psychotherapy for PTSD/Traumatic Grief (BEPP)	(36, 37)	Letter	Ongoing farewell letter
		Dialogue	Imaginary conversation with the deceased
		Ritual	Farewell ritual
Narrative Exposure Therapy (NET)	(38)	Ritual	Light a candle (start of treatment), ending ritual

The research question and Table 1 served as input for the search terms. We collected terms based on these lead publications using the Ovid Citation Analyzer. After that we built a search strategy in PsycINFO (Ovid), which we then adapted to the other databases, and search systems. The whole search strategy for PsycINFO (Ovid) is shown in Table 2.

**Table 2:** Search strategy for PsycINFO (Ovid)^a^.

**#**	**Searches**	**Results**	**Type**
**Search history sorted by search number ascending**			
1	(pcbd or pgd or ((Complex or complicated or prolonged or persist^*^ or traumat^*^ or pathological) adj2 (bereave^*^ or grief or grieving or mourn^*^))).ti,ab.	2,541	Advanced
2	exp Post-traumatic Stress Disorder/or exp acute stress disorder/or exp combat experience/or exp Emotional Trauma/or exp Post-Traumatic Stress/or exp Stress Reactions/or trauma/or exp traumatic neurosis/or (psychotrauma^*^ or Trauma or PTSD or DES^*^NOS or C^*^PTSD or EPCACE or multitrauma or traumatized or traumatized or DTD or “Enduring Personality Change after Catastrophic Experience^*^” or (Stress adj3 disorder^*^) or ((combat or war) adj3 (experience^*^ or disorder^*^ or fatigue or neurosis or neuroses or stress)) or ((Emotional or Complex or chronic or Complicated or Multiple) adj3 Trauma^*^) or (acute adj3 Stress) or ((Stress or Crisis) adj3 Reaction^*^) or ((Post-Traumatic or post-traumatic or Trauma^*^) adj3 (stress or neurosis or neuroses or syndrome^*^ or Disorder^*^ or psychosis or psychoses or distress^*^)) or (Shell adj1 Shock) or (Compassion adj3 Fatigue) or (type adj3 trauma) or (trauma adj2 (stressor adj2 disorders))).ti,ab.	115,582	Advanced
3	exp grief/or exp bereavement/or (bereave^*^ or bereft or grief^*^ or grieving or griever^*^ or mourn^*^ or sorrow^*^ or lament^*^).ti,ab,id.	25,838	Advanced
4	Treatment/or exp Cognitive Behavior Therapy/or exp Behavior Therapy/or exp Psychotherapy/or (treatment^*^ or intervention^*^ or therapy or therapies or therapeutic^*^ or psychotherapy or psychotherapies or psychotherapeutic^*^ or program^*^ or session^*^ or BEPP or BEP-TG or CGT or “complicated grief treatment” or “Finding Meaning in Loss” or cbt or Abreact^*^ or Autosuggesti^*^ or Bibliotherap^*^ or catharsis or countertransferen^*^ or ECT or Hypno^*^ or Jocotherap^*^ or katharsis or logotherapy or mindful^*^ or narcotherapy or psychopharmacotherapy^*^ or Self-Analy^*^ or sociotherap^*^ or transferen^*^ or EMDR or “eye movement desensiti^*^” or CBSM or desensitization or desensitization or “psychiatric somatic therap^*^” or (brief adj3 eclectic) or (psychiatric adj3 (treatment^*^ or intervention^*^ or therap^*^)) or ((anger or assertive^*^ or autogen^*^ or behavio^*^ or milieu or relaxation) adj3 training) or (behavio^*^ adj3 contract^*^) or ((legal or involuntary or psychiatric or psychiatric) adj3 commitment) or ((transact^*^ or behavior^*^) adj3 analysis) or (cognitive adj3 (behavior^*^ or rehabilit^*^)) or (relax^*^ adj3 (method^*^ or Techni^*^)) or (role adj3 play^*^) or ((systematic or psychologic) adj3 desensiti^*^) or ((involuntary or compulsory) adj3 (admission or hospitali^*^)) or (Balint adj3 group^*^) or (behavior^*^ adj3 modification) or ((auto or self) adj3 suggestion) or (free adj3 association) or (anger adj3 management) or (family adj3 psychiatry) or (guided adj3 imagery) or (analytical adj3 psychology) or ((imaginal or vivo) adj3 exposure)).ti,ab.	1,536,756	Advanced
5	exp evidence based practice/or exp Treatment Effectiveness Evaluation/or Clinical Trials/or Mental Health Program Evaluation/or Placebo/or ((evidence adj1 (base^*^ or inform^*^)) or empirical or	2,217,492	Advanced
	cohort or (case and (comparison or referent)) or risk or causation or causal or “odds ratio” or etiol^*^ or etiol^*^ or “natural history” or predict^*^ or prognos^*^ or outcome or course or retrospect^*^ or “clinical trial” or ((singl^*^ or doubl^*^ or trebl^*^ or tripl^*^) and (mask^*^ or blind^*^)) or “latin square” or placebo^*^ or random^*^ or control or controll^*^ or prospectiv^*^ or volunteer^*^ or “research design” or ((comparative or evaluation or follow-up or prospective or cross-over) adj1 stud^*^) or (disability and evaluation^*^) or ((statistical or Probabilistic or Polynomial or “two parameter^*^” or “2 parameter^*^” or Binomial) and (model or models)) or (likelihood and (functions or function or estimat^*^)) or ((linear or loglinear or logistic) and (model or models or regression^*^)) or ((time or risk or risks) and (factor or factors)) or regression^*^ or multivariate or (recover^*^ and (function or functions)) or sensitivit^*^ or “area under curve^*^” or auc or prognos^*^ or placebo^*^ or randomly or randomi^*^ or trial or ((singl^*^ or doubl^*^ or trebl^*^ or tripl^*^) adj3 (blind^*^ or mask^*^ or dummy)) or (control^*^ adj3 (trial^*^ or study or studies or group^*^)) or factorial^*^ or allocat^*^ or assign^*^ or volunteer^*^ or crossover^*^ or “cross over^*^” or (quasi adj5 (experimental or random^*^)) or groups).ti,ab.		
6	(cohort^*^ or longitudinal or prospective or retrospective or timeserie^*^ or followup or (repeated adj1 measure^*^) or (pre adj1 post) or (time adj1 serie^*^) or (follow adj1 up) or (panel adj3 stud^*^)).ti,ab.	344,535	Advanced
7	Literature Review/or Meta Analysis/or Treatment Guidelines/or ((reviews.dt. or (review or “systematic review”).pt.) and systemat^*^.ti,ab.) or (“systematic review” or “systematic literature” or “integrative review” or “integrative literature” or “evidence-based review” or “evidence-based overview” or “evidence-based literature” or “evidence-based survey” or “literature search”).ti,ab. or (“data synthesis” or “evidence synthesis” or “data extraction” or “study selection”).ti,ab. or “cochrane database syst rev.”jn,jx,jw. or (meta-analy^*^ or metaanaly^*^ or metanaly^*^).ti,ab. or meta^*^.dt. or “meta analysis”.pt. or (meta-synthesis or metasynthesis or meta-study or metastudy or metaethnograph^*^ or meta-ethnograph^*^).ti,ab. or hta.ti,ab. or (“health technol assess” or “evid rep technol assess summ”).jn,jx,jw. or “health technology assessment”.ti,ab. or (guideline or framework or manual protocol).ti,ab.	259,425	Advanced
8	(1 or (2 and 3)) and 4 and (5 or 6 or 7)	1,576	Advanced

a *The search strategies in the following search systems were adapted further as follows*.

Ovid Medline: because “trauma” in Medline often refers to physical trauma, these terms were left out of the search in set 2: trauma, multitrauma, traumatized, traumatized, ((Complex or chronic or Complicated or Multiple) adj3 Trauma^*^), (type adj3 trauma).

Embase (Ovid): because “trauma” in Embase often refers to physical trauma, these terms were left out of the search in set 2: trauma, multitrauma, traumatized, traumatized, ((Complex or chronic or Complicated or Multiple) adj3 Trauma^*^), (type adj3 trauma). Also, “PCBD” and “PGD” rendered a lot of irrelevant results and were taken out of set 1.

*PTSDpubs: the elaborate search strategy caused PTSDpubs to jam, so we pruned the search strategy strongly, using mainly subject terms where possible*.

*TRIP: our free version does not support complex search strategies, so we used a simplified search string*.

*NICE, ICRP, OpenGrey: these search systems do not support complex search strategies, so we used a simplified search string*.

The search terms were grouped into clusters (see Table 2 for the set numbers and the details of the search terms): complex grief (set 1), grief in the context of traumatic stress (sets 2 and 3), psychotherapy and behavioral therapy (set 4), evidence based practice, program evaluation, clinical trials (set 5), longitudinal studies (set 6), aggregated evidence: systematic reviews, meta-analyses, guidelines (set 7). These clusters were combined using Boolean operators. The search results were imported in Endnote and deduplicated using the method outlined in Bramer et al. (39). Table 3 shows the number of references retrieved in each search system, the number of duplicates, and thus the new articles collected. These articles were then screened for inclusion in this review.

**Table 3:** Retrieved, duplicate, and unique items from each search system.

**Database**	**Number retrieved**	**External dups**	**New articles added**
PsycInfo (Ovid)	1,576	546	1,030
Ovid Medline	1,507	682	825
Embase (Ovid)	1,185	126	1,059
Ovid Evidence Based Medicine Reviews	358	214	144
PTSDpubs	160	11	149
TRIP database	78	4	74
NICE	1,143	50	1,093
ICTRP	40	6	34
Open Gray	2	0	2
**Totals**	6,049	1,639	4,410

### Screening and Selection

**Figure 1:**
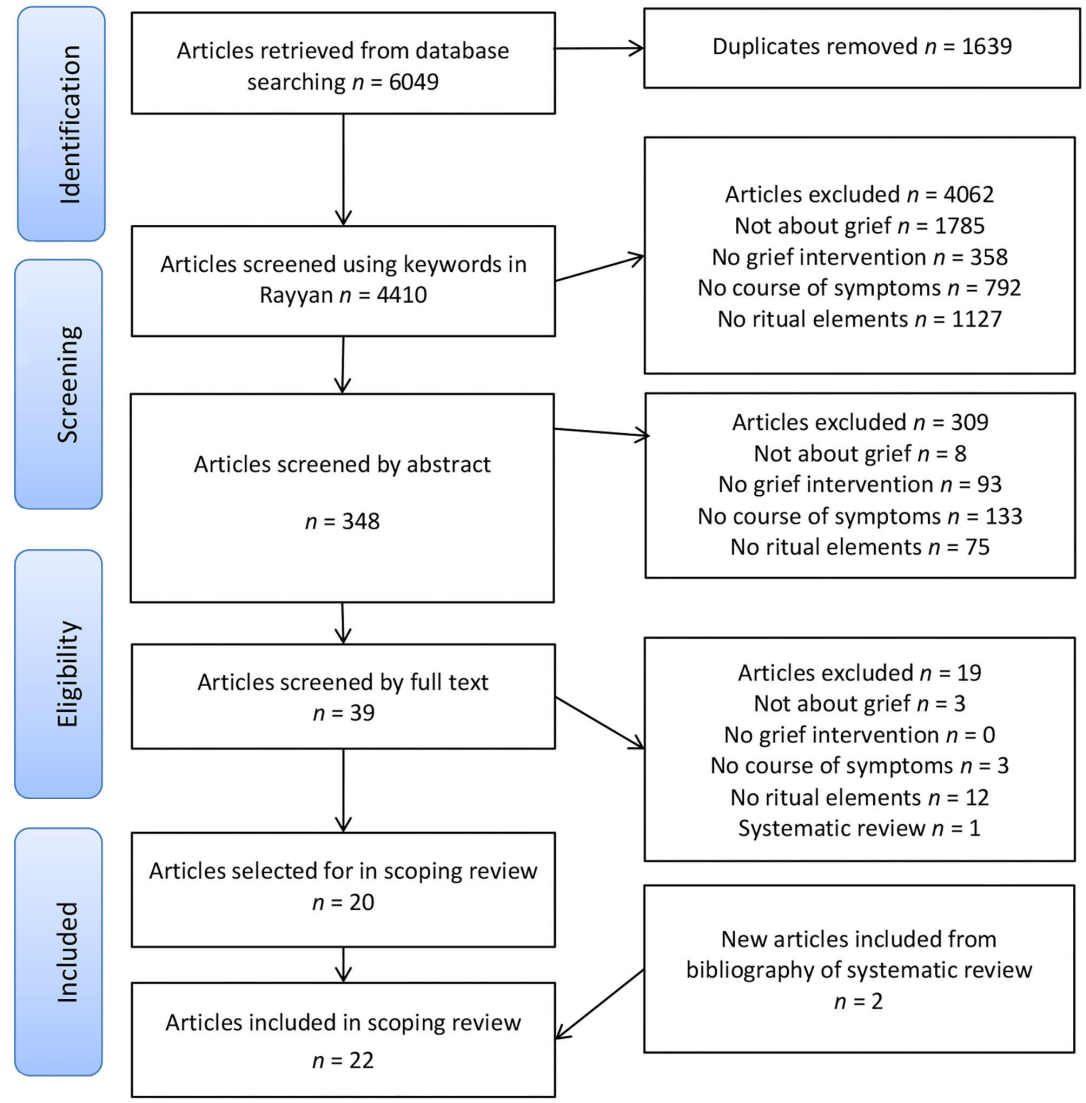
Flowchart of the retrieval, deduplication, exclusion, and inclusion of articles.

In order to screen and select articles for this review, we used Rayyan (a web-based program for systematic reviews), see Ouzanni et al. (40). Four inclusion criteria were used for the screening and selection (see Table 4). A reference was selected if (1) it referred to grief, bereavement or loss, (2) described a grief intervention, (3) measured course of symptoms in relation to the grief intervention, and (4) included ritual elements. Ritual elements were operationalized as (a) a form of symbolic communication and/or (b) use of ritualized action, objects or language and/or (c) included a reference to religious ritual (e.g., prayer, meditation) *and* (d) addressed the grief or loss experience. In this way, with each step, references not meeting one of these four criteria were excluded—as shown in Figure 1. These inclusion criteria were translated into keywords in Rayyan (see Table 5 for complete overview of Rayyan keywords). Four thousand four hundred and ten references were screened on the basis of the four inclusion criteria (Table 4).

**Table 4:** Inclusion criteria for references.

**Criterion**	**Description**
1	-Refers to grief, bereavement, or loss
2	-Describes grief intervention(s)
3	-Describes course of symptoms after grief intervention (quantitative measurement)
4	-Intervention includes ritual elements

**Table 5:** Overview keywords used for screening in Rayyan.

	**Screening process**	**Used keywords in Rayyan**
Starting point	Outcomes systematic search (*n* = 4410)	
Criterion 1	All references with keyword grief and loss (*n* = 2625)	Bereaved Bereavement Grief Griever Grieving Loss Mourn Mourner Mourners Mourning Post traumatic Posttraumatic Post-traumatic PTSD Sorrow Stress Trauma Traumas Traumatic Traumatised Traumatized
Criterion 2	All references with grief intervention (*n* = 2267)	Counseling Counselling Intervention Interventions Psychotherapy Psychotherapies Therapeutic Therapy Therapies Treating Treatment Treatments
Criterion 3	All references with measurement of symptoms (*n* = 1475)	Baseline Compare Compared Comparison Condition Conditions Control Controlled Follow-up Improvement Improvements Measurement Measurements Measuring Outcome Pretest Pre-test Pretesting Pre-testing Posttest Post-test Posttesting Post-testing Posttreatment
		Post-treatment Quantitative Randomised Randomized Randomly RCT Symptoms Test Testing Trial Trials
Criterion 4	All references with possibly ritual elements in grief intervention (*n* = 348)	Brief eclectic Candle Ceremonial Ceremony Farewell Goodbye Imaginary conversation Integrative Meaning Meaning-making Memento Memorial Memories Mindfulness based Mindfulness-based Narrative Narrative exposure Object Remembering Rite Ritual Ritualising Ritualizing Sense Sense-making Symbol Symbolic Transformation Transformative Transition Transitional
	Next step	348 abstracts were read to check inclusion criteria 1-4

The first inclusion criterion, which refers to grief, trauma, bereavement or loss (keywords such as *grief, trauma, bereavement, bereaved, post-traumatic, post-traumatic stress, complicated grief, traumatic grief, bereaved, mourn, mourning, or loss*), resulted in 2,625 references. The second criterion included references that describe a grief intervention (keywords such as *psychotherapy, therapy, intervention, treatment)* resulted in 2,267 references. The third criterion included references that contain information about the course of symptoms after the grief intervention (keywords such as *pretest, posttest, pre-test, post-test, test, quantitative, measurement, testing, symptoms, trial, randomized, randomized, RTC, controlled, control groups, randomly allocated, controlled study*), resulting in 1,475 references. The fourth inclusion criterion referred to articles that included ritual elements in the grief intervention (keywords such as *ritual, symbol, memorial, writing, imaginary conversation, candle, metaphor*), which resulted in a final selection of 348 references.

The abstracts of these 348 references were read and selected on the basis of the four inclusion criteria (Table 4). We selected references on the basis of criterion 1 (including *n* = 340 references), criterion 2 (*n* = 247). Criterion 3 (*n* = 114) and criterion 4 (arriving at 39 references). Again, this led to the exclusion of references not meeting all of the criteria (*n* = 309). The full texts of the remaining 39 references were screened excluding 19 on the basis of criterion 1 (*n* = 3), criterion 3 (*n* = 3) and criterion 4 (*n* = 12). One of these full-texts articles, a systematic review of evidence-based grief interventions after homicide (41), yielded two more relevant studies on grief therapy and ritual elements, which were included in the final results. Not including this systematic review, we arrived at a final overview of 22 studies (see Appendix 1).

### Charting the Data

The included 22 studies are summarized in Appendix 1 describing the name of the intervention, the reference that reported the intervention, a description of the intervention and the used ritual elements, the studied population, the type of evidence in terms of study design and data collection, effect on grief, and level of evidence (42). The CEBM levels of evidence have been chosen for this study due to the clear, transparent and broad use of this categorization of evidence levels (30, 42). Level 1 refers to “systematic reviews of randomized trials or *n*-of-1 trials.” Level 2 refers to “randomized trial or observational study with dramatic effect.” Level 3 are “non-randomized controlled cohort/follow-up” studies. Level 4 refers to “case-series, case-control studies, or historically controlled studies” and level 5 is based on “mechanism-based reasoning.” The levels of evidence may be graded down on the basis of study quality, imprecision or effect size and the like. Not all articles included an in-depth description of the grief intervention, but referred to additional articles or manuals, which were used for a more in-depth description of ritual elements.

## Results

### General Description of Grief Therapies

The selected studies reported different populations with prolonged or traumatic grief, such as violent loss (e.g., homicide), genocide and war trauma, as well as more general grief reactions, such as elderly bereaved losing a spouse with persistent grief distress, professionals (e.g., police or hospice workers), loss due to a missing person or perinatal loss. The studies were conducted in different countries, such as United States (*n* = 7), The Netherlands (*n* = 4) (one study with refugee population), Portugal (*n* = 2), Rwanda (*n* = 2), Germany (*n* = 2), India (*n* = 2), Japan (*n* = 1), Denmark (*n* = 1), and Iran (*n* = 1) (with Afghan refugee population). The studies found for this review were published between 2009 and 2019. All studies reported participants being selected in outpatient contexts. Eight of the reported interventions are individual [*ATTEND-model, Cognitive Narrative Psychotherapy, Complicated Grief Treatment, Writing assignments (Pennebaker's model), Traumatic Grief Treatment Program, Narrative Exposure Therapy (NET), Integrative Cognitive Behavioral Therapy for Complicated Grief (CG-CBT), Cognitive Behavioral Therapy (CBT) with mindfulness]*, while nine are group interventions [*Mindful-based Cognitive Therapy (MBCT), Mustard-Seed Project, Restorative Retelling, TOZI Healing Retreat, Brief Eclectic Psychotherapy for PTSD/Post-traumatic grief (BEPP), Brief Eclectic Psychotherapy for Traumatic Grief (BEP-TG), a Single Session Music Therapy, Writing for Recovery, Mindfulness-based Stress Reduction (MBSR)*]. The described treatments vary in time span (from 15 min to 3.5 h) and length [from a single session to 20 sessions with additionally 5 optional sessions *(CB-CBT*)]. Some treatments are weekly sessions, while a few are offered 2 or 3 days in a row, in some cases with overnight housing (*Mustard Seed Project and TOZI Healing retreat)*.

### Ritual Elements in Grief Therapies

The grief interventions reveal different ritual elements, ranging from meditation, symbolic communication with the deceased or an imaginary friend, to metaphorization and other types of symbolic expression (e.g., silence at the beginning or end of a session, writing assignments, the use of religious texts or poems). Some interventions consist of one specific ritual element (e.g., imaginal conversation with deceased, writing letter to imaginary friend, metaphorization of loss), while others have more or even include a complete ceremony at the end of the treatment or intervention (e.g., drumming ceremony, visiting the funeral with the therapist, visiting the grave, having a dialogue with the deceased, having a commemorative ceremony). The degree of ritual use thus differs in the different grief interventions. Some interventions make use of specific body techniques, such as sitting or meditation, while others use specific objects, such as memorial objects that are linked to the deceased or the loss. Sometimes these mementos are used in a commemorative presentation or ceremony at the end of the treatment. Some interventions have a (optional) farewell ritual that the client can conduct privately, such as with family or other significant people.

The involvement of the therapist in the ritual elements differs and is not always clear from the descriptions. In some individual ritual interventions or home assignments clients are asked to conduct the ritual privately (such as CBT with mindfulness). Clients are asked to read the mindfulness exercise instructions online or follow them on CD (43). In the Mustard-Seed project (44) the therapist works together with a Zen-Monk and they both guide the sessions. In the ATTEND model, the therapist is asked to attune to the client and focus on one's own presence and self-compassion, for instance by meditating after a workday or doing some physical exercise (45, 46). In the group sessions and ceremonial rituals (e.g., commemoration or farewell ritual) it is not clear from the described articles or intervention manuals whether the therapist leads the ceremony. In the BEP-TG manual [see Smid et al. (36)] it is explicitly written that the therapist is not present during the farewell ritual and that the patient designs her/his own farewell ritual. The final phase of the Integrative Cognitive Behavioral Therapy is described as: “patients decide what kind of memento/ritual they will dedicate to their loved ones” [(34), p. 7], which points at the ritual being conducted without the therapist being present.

### Evidence Levels for Grief Therapies With Ritual Elements

As can be seen in Appendix 1, 8 studies were categorized level 2 of evidence (RCT with significant symptom reduction), 11 studies were categorized as level 3 evidence (non-randomized controlled cohort/follow-up study) and three studies were categorized as level 4 due to no significant result of the treatment [one RCT with no significant effect; (47), two pre/post-tests with no significant result due to small sample; (48, 49)]. All measured effects were reported for the entire treatment, with the exception of one observational study that measured a significant separate effect on the ritual element (farewell ritual) (50).

The different studies reveal symptom reduction on different measurements (see Appendix 1), such as *grief* [e.g., (51–55)], *depression* [e.g., (46, 56), PTSD (57, 58)], *distress due to trauma* [e.g., (46, 59)], *despair and panic behavior* (44), *intrusions and avoidance* (55), *emotional loneliness* (60) and *emotional numbing* (59). Moreover, some studies reported an improvement in *working memory* (56), *positive mood* (60), *personal growth and meaning integration* (44).

## Discussion

This scoping review of ritual elements in evidence-informed grief interventions for prolonged grief identified 22 studies revealing a variety of ritual elements for different types of grief and loss experiences. Rituals are used in various therapy interventions for prolonged and traumatic grief, making up a sizable part of grief treatment. Although three studies did not show significant treatment effects (47–49), 19 studies revealed significant treatment effects on symptom reduction. It is important to note that, with the exception of Smid et al. (50), the reported effects concerned the entire treatment and not only the ritual element(s). This means that the studies reported here measured effects in terms of reductions in grief or other symptoms after completion of the full treatment, not only the ritual element.

The described ritual elements show different forms of verbal symbolic expression of one's personal experiences (such as finding a metaphor for one's loss) [e.g., (52, 59)] and symbolic interaction with the deceased or imaginary friend (such as an imaginative conversation with the deceased or writing a letter to deceased loved one) [e.g., (60–62)], as well as non-verbal symbolic expressions (such as lighting a candle, visiting the grave, burning a letter, choosing objects and images to represent deceased) [e.g., (38, 46, 50, 53, 61)]. A focus on attention and bodily posture has been found in some of the interventions, where the use of silence, meditation, sitting in a circle, breathing or yoga exercises are implemented in the therapy (44, 46, 56, 63, 64).

Rituals have positive characteristics for dealing with prolonged grief as they are structured: the action is time limited (12) and the ritual symbolically and aesthetically translates the grief experience into ritual form (10, 24). Rituals take place within a symbolic reality, which gives some emotional distance to the actual experience, but at the same time creates space to feel and express emotions (24, 65, 66). By choosing objects or symbols that represent the deceased or conducting a memorial during therapy, grief emotions are channelized through the ritual form. The use of symbols and symbolic actions (e.g., having a moment of silence, lighting a candle or listening to a special music piece) helps to stay attentive and feel emotions. Emotions are intense, but not overwhelming as the time frame is limited and the actions are ritually structured. The symbolic expression and interaction help to feel emotions, but because of the aesthetic translation the emotional expression remains at some distance [see for a discussion on aesthetic distance, (24, 65, 66)]. The strength of ritual and ritual interventions lies in the use of symbolic expression and interaction. For example, while we know that we are unable to literally talk to the deceased, we do it anyway. The focus in the ritual element, such as an imaginary conversation with the deceased, is on care and attention toward the deceased. When one was unable to take care of the loved one in the past, this can be reimagined in one's thoughts or symbolic action. The person uses her imagination in connecting with the loved one or to reimage the past as one would want it to happen (“rescripting”) (67). The imagined world in ritual feels authentic and real (27), which makes the ritual emotionally intense and at the same time gives an opportunity to symbolically act out something that could not be done before. Moreover, from previous research was shown that specific ritual characteristics, such as the number of procedural steps, repetition, procedural specificity (such as time), and transcendental notions (a religious icon) are related to a higher evaluation of ritual efficacy (17). The here studied ritual elements might be furthermore distinguished in terms of these specific ritual characteristics. Some of the here described ritual include more procedural steps, mostly the ceremonies at the end of the treatment, while others involve one ritual element (e.g., write a letter to imagined person). Writing a letter, however, has also in itself procedural steps, which might be of importance to notice in grief treatment. For clinical practitioners it is thus of importance to know that procedural steps and their specificity is relevant for ritual efficacy. It is not mere routine, but significant for the emotional and cognitive processing during the ritual (21, 29).

Some of the here described rituals are conduced individually (46, 51, 52, 57–59, 62) or alone as a home assignment (60), or both (48). Other rituals take place in a group setting with other fellow bereaved present (44, 53, 55, 56, 63, 64, 68) and sometimes include a farewell ceremony at the end of the treatment [e.g., (47, 49)]. In one farewell ritual family members or other loved ones can also participate (50).

The social aspect of ritual and the sense of community during ritual has been argued to be one of the key functions of rituals in our society (25, 69, 70). Conducting ritual action together, especially when the ritual is associated with pain and grief, is argued to create a sense of cohesion and solidarity (25). Having others to share one's grief with, as well as going through an emotional ritual together is experienced as comforting. Moreover, Boyer and Liénard (21) argue that cultural rituals, as also mentioned earlier, work on the basis of cohesion and identity fusion [see also Whitehouse and Lanman (25)] as well as, by triggering “activation of motivational systems and cognitive processes that are present in humans for evolutionary reasons” [(29), p. 825]. Individual rituals are argued to be effective due to the involvement of the “hazard-precaution system,” which is activated in ritualized behavior. This cognitive system is alerted when potentional dangers cross one's thoughts, such as contamination and contagion, harm to one's offspring or acts that lead to social exclusion (21, 29). Different from actual danger or fear reactions, this precaution system is often activated in phase of social and emotional transition, such as in pregnancy and caring for an infant, as well as the death of a relative [(21), p. 606]. The authors strongly lean on research on compulsory ritualized action (OCD) and argue that “ritualized behavior” or “action ritualization” temporarily suppresses undesirable thoughts and “momentary overload” or “swamp” working memory [(21), p. 605], as well as require a “high degree of cognitive control” [(21), p. 607]. Therefore, ritualized behavior is the opposite of routinized behavior, as in routines cognitive and attentive load is low [(29), p. 824]. It is thus of importance to notice that rituals psychologically differ from routines. The steps and sequences in ritual procedure are of uttermost importance. As Seligman et al. (71) write “it is the framing of the actions, not the actions themselves, that make them rituals” (p. 5).

The ritual elements that were found in this study reveal different types of rituals. Sas and Coman (12) categorized three types of grief rituals in therapy: (1) honoring rituals, (2) letting go rituals, and (3) self-transformation rituals. In the here described ritual interventions, we can see elements of all three. In some interventions the deceased is honored through symbolic expression, for instance choosing mementos or memorial objects to represent the deceased [e.g., (50, 55, 61)]. The farewell ritual in the BEPP intervention is described in the manual as: “the ritual serves to leave the traumatic experience behind. The purpose is not to forget the experience, but to give it a place in the client's personal life history” [(72), p. 32]. This description refers to ritual for letting go but might also contain elements of honoring the deceased. Rituals of continuity or honoring emphasize that the deceased is still remembered. Rituals of transition affirm that one has entered a new place in one's journey through grief. The “healing drum session” during the TOZI-retreat might be considered a transition ritual (49), as well as the laying out of the symbolic lifeline in narrative exposure therapy (57) or burning the “angry” letter in the BEP-TG treatment (61).

In the literature, there is also discussion of rituals of reconciliation that either ask for or extend forgiveness and rituals of affirmation provide ways to acknowledge legacies or to say thanks (73). A key function of death rituals is to provide structured ways to mourn and express grief, and death rituals may serve important social, cultural, and psychological functions that foster adaptation to loss (3, 10). While some ritual elements might serve more than one function, it is suggested here that the different ritual interventions are used with different functions and intentions in the various grief treatments.

Some ritual elements focus more on embodied states in terms of meditation, sitting, walking or breathing (in silence), revealing what in the literature has been discussed as “body techniques” in ritual (74). Body techniques are culturally learned ways of “using” the body in a specific way, such as using tools to eat or religious prayer or meditation. In cases of traumatic loss, bereaved often use non-verbal communication to express their emotions, such as touching their face, staring (in front of them), throwing their hands in the air, touching or hugging the body of the deceased or the coffin. In the here found grief interventions, body techniques are used to bring the person into a specific embodied state. The use of silence might help to focus on one's bodily sensations and possibly inner contemplation. Body techniques are an important part of rituals, more generally, because they help to stay focused during the ritual (e.g., remain in silence or focus attention toward a central ritual performance) and to embody the aspired action (e.g., connect with the divine, pray for the deceased or clap during a graduation ceremony). Body techniques are used in various treatments for prolonged and traumatic grief, because they help to feel emotions and sensory input within a safe environment. Other ritual elements focus on addressing painful emotions or sensations, such as re-enacting the dying scene, finding a metaphor for loss, writing an “angry” letter to the perpetrator. Rituals in grief therapy, thus, are helpful due to different processes, such as expressing various emotions (thankfulness, care for the deceased, but also feelings of guilt, and asking for forgiveness), as well as creating a bond with the deceased and other bereaved, which perhaps was before the treatment ambivalent or problematic. Rituals structure internal and external processes through ritualized action (21, 24).

The cultural dimension of grief treatment needs also to be discussed here in relation to the different grief treatments, especially in today's globalized societies. Rituals have an important role in dealing with grief across cultures. Culture relates to ritual customs, as well as how we deal with grief. A cross-cultural study comparing data on mortuary rites from 57 cultures across the world revealed that in 93% of the cases kin is exposed to and in 89.5% of the cases has contact with the dead body (75). In a majority of cultures (71.9%) a moderate intimacy with the dead body is reported and in some a low (8.8%) or high (7%) intimacy. The authors did not find significant differences in the degree to which the body is threated on the basis of these criteria between different continents (e.g., Asia, Afrika, Europe). They furthermore argue that researchers (in this case anthropologists) are required to interpret the meanings of the specific ritual acts across cultures, as mortuary rituals differ across cultures. Research has suggested psychological benefits of mourning rituals, such as regained feelings of control and social support (76, 77), which might be the case in the here described ritual interventions in prolonged and traumatic grief treatment.

In specific grief contexts, such as following the loss of loved ones due to a disaster, immigrant ethnic minority group members have been found to endorse more persistent symptoms than natives (78). Different explanations for increased mental health symptoms following loss of loved ones and migration have been suggested that involve a lack of rituals. Firstly, *ritual omission* may result from the impossibility of performing culturally appropriate rituals (79) that may be due to migration and/or traumatic circumstances of the death. Secondly, *cultural incongruity* (80) may occur, a mismatch between cultural customs in the host country and cultural traditions of immigrants that may prevent death rituals from fostering adaptation to loss. Cultural norms can prevent the loss of a loved one from being openly acknowledged, publicly mourned, or socially supported, such as in disenfranchised grief (81). Disenfranchisement in grief is found when the grief is not socially acknowledged. It occurs when the mourner or deceased are not considered significant enough or are even condemned (e.g., the person is considered at the wrong side during the war), the relationship with the deceased (e.g., extra-marital relationships), the circumstances of the death (e.g., self-inflicted death), or other aspects of the loss that are not socially acknowledged. Rituals can be ways to make disenfranchised grief visible and create a way to cope with the grief (82).

Culturally appropriate rituals need to be taken into account during end-of-life care. A study examining the potential benefits of the end-of-life informal caregiving, communication, and ritualized behaviors in adaptation to the conjugal bereavement across two different cultural contexts, France and Togo (*n* = 235), showed that postloss growth in Togolese bereaved was fostered by end-of-life communication with the dying and the performance of ritualized behaviors. In French bereaved individuals, experiencing more intimate communication with the dying spouse was associated with a higher level of postloss growth. The authors concluded that informal caregiving to the dying, communication with the dying, and ritual support need to be promoted as integrated components of end-of-life care (7).

### Study Strengths and Limitations

This study has shown that various ritual elements are used in different grief interventions and most of the reported studies reveal evidence toward the effectiveness of the treatment. However, as most effects were studied for the entire treatment, a separate effect for the ritual elements needs further investigation. Furthermore, measuring and comparing effects for different types of therapy have some validity issues that need to be mentioned (83). For instance, the higher stressful grief symptoms in general within a population before the therapy, the higher these symptoms will be after the treatment also [(83), p. 71]. Furthermore, there are “patient factors” that increase symptom reduction due to motivation and readiness for change, economic and social resources, which are not necessary linked to a specific treatment [(83), p. 71]. Another difficulty in comparing the “relative efficacy” of different treatments lies in the debate around specific “ingredients” in therapy [(83), p. 114]. Common factors might be involved in the effectiveness of psychotherapies in general, including the treatment alliance, genuineness, patient expectations, and therapist empathy. A final difficulty with identifying ritual elements in existing therapy is that in most studies the intervention is not described in detail, as published data do not routinely include the intervention manual. This might have led to the inadvertent exclusion of some articles that actually did include a grief intervention with ritual elements.

### Implications for Research and Practice

A focus on separate effects for different parts of the treatment can be included in future research, as well as adding qualitative measurement, such as open questions about what clients appreciated most during the therapy and if possible, whether or not the ritual elements were remembered and how they were evaluated. As rituals are also present within one's cultural upbringing and some of the rituals refer to traditional ritual actions (e.g., meditation) or include the reading of religious or spiritual texts, it would be noteworthy to learn more about the cultural associations that clients have with these elements. What kind of thoughts and meanings do bereaved experience during the rituals? How are the rituals during the intervention linked to the rituals that one knows from one's own cultural background? More research is needed to focus more in these questions and we hope by providing this scoping review that we can add to the discussion on rituals in grief therapy.

The here reported studies were conducted in countries across the world. In some studies, the original grief intervention was translated and explicitly adjusted to the specific cultural context (57, 58, 63, 64). Making use of rituals in therapy asks for more focus on and discussion about the role of culture in grief therapy. For mental healthcare providers who serve culturally diverse clients, cultural assessment of bereavement and grief is needed for a comprehensive evaluation of grief-related psychopathology and for negotiating appropriate treatment. The Bereavement and Grief Cultural Formulation Interview (BG-CFI) comprises a set of brief, person-centered, and open-ended questions (84), included as a supplementary module to the DSM-5 Cultural Formulation Interview (85). The BG-CFI assesses cultural traditions related to death, bereavement, and mourning as well as help seeking and coping. Using these questions, the clinician may explore cultural aspects of bereavement and grief in patients seeking mental health care following the loss of loved ones in order to enhance understanding as well as tailor interventions to alleviate distress (84).

## Data Availability Statement

The original contributions presented in the study are included in the article/Supplementary Material, further inquiries can be directed to the corresponding author/s.

## Author Contributions

JW and GS have initiated this research and formulated the research question and worked out the research design and selected the studies for this research. JL has provided the source data by conducting the search and has written the method section. JW has reported on the outcomes with comments from JL and GS. All authors contributed to the article and approved the submitted version.

## Conflict of Interest

The authors declare that the research was conducted in the absence of any commercial or financial relationships that could be construed as a potential conflict of interest.

## References

[1] A.P.A Diagnostic and Statistical Manual of Mental Disorders, 5th ed (DSM-5) Washington, DC: A.P.A (2013).

[2] World Health Organization International Classification of Diseases, 11th Revision (ICD-11). Geneva: WHO (2018).

[3] Cacciatore J DeFrain J The world of bereavement: cultural perspectives on death in families. In: International and Cultural Psychology. Cham; Heidelberg: Springer International Publishing (2015). 10.1007/978-3-319-13945-6

[4] Robben ACGM A companion to the anthropology of death. In: Wiley Blackwell Companions to Anthropology. Hoboken, NJ, Oxford: Wiley (2018). 10.1002/9781119222422

[5] Selin H Rakoff RM Death across cultures: death and dying in non-western cultures. In: Science Across Cultures: The History of Non-Western Science. Cham: Springer International Publishing (2019). 10.1007/978-3-030-18826-9

[6] Hertz R Death and the right hand. In: A Contribution to the Study of the Collective Representation of Death. (tr. Rodney and Claudia Needham). London: Cohen & West (1960).

[7] Kokou-Kpolou CK Moukouta CS Sani L McIntee SE Cénat JM Awesso A. A mixed methods approach of end-of-life care, social rites, and bereavement outcomes: a transnational perspective. Cult Med Psychiatry. (2020) 44:501–23. 10.1007/s11013-020-09669-3 32124133

[8] Robben ACGM (ed.) (2004). Death, Mourning and Burial. Malden, MA: Blackwell Publishing.

[9] Walter T. Why different countries manage death differently: a comparative analysis of modern urban societies. Br J Sociol. (2012) 63:123–45. 10.1111/j.1468-4446.2011.01396.x 22404392

[10] Romanoff B Terenzio M. Rituals and the grieving process. Death Stud. (1998) 22:697–711. 10.1080/074811898201227 10346698

[11] Goodwyn ED Healing Symbols in Psychotherapy: A Ritual Approach. New York, NY: Routledge (2016). 10.4324/9781315651811

[12] Sas C Coman A. Designing personal grief rituals: an analysis of symbolic objects and actions. Death Stud. (2016) 49:558–69. 10.1080/07481187.2016.1188868 27603436

[13] Sas C Whittaker S Zimmerman J Design for rituals of letting go: an embodiement perspective on disposal practices informed grief therapy. ACM Trans Comput Hum Interact. (2016) 23:21–37. 10.1145/2926714

[14] Neimeyer RA Meaning reconstruction in the wake of loss: evolution of a research program. Behav Change. (2016) 33:65–79. 10.1017/bec.2016.4

[15] Hart O van der Goossens FA Leave-taking rituals in mourning therapy. Isr J Psychiatry Relat Sci. (1987) 24:87–98. 2450853

[16] Grimes RL The Craft of Ritual Studies. Oxford: Oxford University Press (2014). 10.1093/acprof:oso/9780195301427.001.0001

[17] Legare CH Souza AL. Evaluating ritual efficacy: evidence from the supernatural. Cognition. (2012) 124:1–15. 10.1016/j.cognition.2012.03.004 22520061

[18] Humphrey C Laidlaw J The Archetypal Actions of Ritual: A Theory of Ritual Illustrated by the Jain Rite of Worship. Oxford: Clarendon Press (1994).

[19] Brooks AW Schroeder J Risen JL Gino F Galinsky AD Norton MI Don't stop believing: rituals improve perfromance by decreasing anxiety. Organ Behav Hum Decis Process. (2016) 137:71–85. 10.1016/j.obhdp.2016.07.004

[20] Hobson NM Schroeder J Risen JL Xygalatas D Inzlicht M. The psychology of rituals: an integrative review and process-based framework. Pers Soc Psychol Rev. (2017) 22:260–84. 10.2139/ssrn.2944235 29130838

[21] Boyer P Liénard P. Why ritualized behavior? Precaution systems and actions parsing in developmental, pathological and cultural rituals. Behav Brain Sci. (2006) 29:595–650. 10.1017/S0140525X06009332 17918647

[22] Quack J Sax WS Introduction: the efficacy of rituals. J Rit Stud. (2010) 5–12.

[23] Schilbrack K (ed.). Thinking Through Rituals. Philosophical Perspectives. New York, NY: Routledge (2004). 10.4324/9780203644416

[24] Wojtkowiak J Towards a psychology of ritual: A theoretical framework of transformative ritual in a globalising world. Cult Psychol. (2018) 24:460–76. 10.1177/1354067X18763797

[25] Whitehouse H Lanman JA The ties that bind us: ritual, fusion and identification. Curr Anthropol. (2014) 55:674–95. 10.1086/678698

[26] Rappaport RA Ritual and Religion in the Making of Humanity. Cambridge: Cambridge University Press (1999). 10.1017/CBO9780511814686

[27] Geertz C The Interpretation of Cultures. Selected Essays by Clifford Geertz. New York, NY: Basic Books, Inc., Publishers (1973).

[28] Goodwyn ED The end of all tears: a dynamic interdisciplinary analysis of mourning and complicated grief with suggested applications for clinicians. J Spirit Ment Health. (2015) 17:239–66. 10.1080/19349637.2015.1047919

[29] Liénard P Boyer P Whence collective rituals? A cultural selection model of ritualized behavior. Am Anthropol. (2006) 108:814–27. 10.1525/aa.2006.108.4.814

[30] Howick J Chalmers I Glasziou P Greenhalgh T Heneghan C Liberati A Explanation of the 2011 Oxford Centre for Evidence-Based Medicine (OCEBM) Levels of Evidence (Background Document). Oxford Centre for Evidence-Based Medicine (2011). Available online at: http://www.cebm.net/index.aspx?o=5653

[31] Arksey H O'Malley L Scoping studies: towards a methodological framework. Int J Soc Res Methodol. (2005) 8:19–32. 10.1080/1364557032000119616

[32] Colhoun HL Levac D O'Brian KK Straus S Tricco AC Perrier L. Scoping reviews: time for clarity in definition, methods, and reporting. J Clin Epidemiol. (2014) 67:1291–94. 10.1016/j.jclinepi.2014.03.013 25034198

[33] Shear MK Frank E Houck PR Reynolds CF. Treatment of complicated grief: a randomized controlled trial. JAMA J Am Med Assoc. (2005) 293:2601–8. 10.1001/jama.293.21.2601 15928281 PMC5953417

[34] Rosner R Pfoh G Kotoucìova M. Treatment of complicated grief. Eur J Psychotraumatol. (2011) 2:7995. 10.3402/ejpt.v2i0.7995 22893810 PMC3402114

[35] Wagner B Knaevelsrud C Maercker A. Internet-based cognitive-behavioral therapy for complicated grief: a randomized controlled trial. Death Stud. (2006) 30:429–53. 10.1080/07481180600614385 16610157

[36] Smid GE Kleber RJ de la Rie SM Bos JBA Gersons BPR Boelen PA. Brief eclectic psychotherapy for traumatic grief (BEP-TG): toward integrated treatment of symptoms related to traumatic loss. Eur J Psychotraumatol. (2015) 6:27324. 10.3402/ejpt.v6.27324 26154434 PMC4495623

[37] Gersons BPR Carlier IVE Lamberts RD van der Kolk BA. Randomized clinical trial of brief eclectic psychotherapy for police officers with posttraumatic stress disorder. J Trauma Stress. (2000) 13:333–47. 10.1023/A:1007793803627 10838679

[38] Schauer M Neuner F Elbert T Narrative Exposure Therapy: A Short-Term Treatment for Traumatic Stress Disorders. Göttingen: Hogrefe Publishing (2011).

[39] Bramer WM Giustini D de Jonge GB Holland L Bekhuis T. De-duplication of database search results for systematic reviews in endnote. J Med Libr Assoc. (2016) 104:240–3. 10.3163/1536-5050.104.3.014 27366130 PMC4915647

[40] Ouzanni M Hammady H Fedorowicz Z Elmagarmid A. Rayyan-a web and mobile app for systematic reviews. Syst Rev. (2016) 5:210–20. 10.1186/s13643-016-0384-4 27919275 PMC5139140

[41] Alves-Costa F Hamilton-Giachritsis C Christie H van Denderen M Halligan S. Psychological interventions for individuals bereaved by homicide: a systematic review. Trauma Violence Abuse. (2019). 10.1177/1524838019881716. [Epub ahead of print]. 31640488

[42] Howick J Chalmers I Glasziou P Greenhalgh T Heneghan C Liberati A The 2011 Oxford CEBM Evidence Levels Of Evidence (Introductory Document). Oxford Centre for Evidence-Based Medicine (2011). Available online at: http://www.cebm.net/index.aspx?o=5653

[43] Lenferink LJM De Keijser J Boelen PA Living with missing. (translated from Dutch: Leven met vermissing.) (2015). Retrieved from: https://www.zoekhonden.com/wp-content/uploads/2018/06/Werkboek-Leven-met-Vermissing.pdf

[44] Neimeyer RA Young-Eisendrath P. Assessing a buddhist treatment for bereavement and loss: the mustard seed project. Death Stud. (2015) 39:263–73. 10.1080/07481187.2014.937973 25365540

[45] Cacciatore J Flint M. ATTEND: Towards a mindfulness-based bereavement care model. Death Stud. (2012) 36:61–82. 10.1080/07481187.2011.591275 24567995

[46] Thieleman K Cacciatore J Wonch Hill P Traumatic bereavement and mindfulness: a preliminary study of mental health outcomes using the ATTEND model. Clin Soc Work J. (2014) 42:260–8. 10.1007/s10615-014-0491-4

[47] Wlodarczyk NM The Effect of a Single Session Music Therapy Group Interventions for Grief Resolution. Tallahassee, FL: Florida State University (2010).

[48] Lenferink LJM De Keijser J Boelen PA Cognitive behavioral therapy and mindfulness for relatives of missing persons: a pilot study. BMC Pilot Feasibility Stud. (2019) 5:93 10.1186/s40814-019-0472-z PMC6642737 31363418

[49] Tuck I Baliko B Schubert CM Anderson L. A pilot study of a weekend retreat intervention for family survivors of homicide. West J Nurs Res. (2012) 34:766–94. 10.1177/0193945912443011 22566289

[50] Smid GE van der Meer CAI Olff M Nijdam MJ. Predictors of outcome and residual symptoms following trauma-focused psychotherapy in police officers with posttraumatic stress disorder. J Trauma Stress. (2018) 31:764–74. 10.1002/jts.22328 30338583

[51] Asukai N Tsuruta N Saito A. Pilot study on traumatic grief treatment program for Japanese women bereaved by violent death. J Trauma Stress. (2011) 24:470–3. 10.1002/jts.20662 21780192

[52] Barbosa V Sa M Carlos Rocha J. Randomised controlled trial of a cognitive narrative intervention for complicated grief in widowhood. Aging Ment Health. (2014) 18:354–62. 10.1080/13607863.2013.833164 24073815

[53] Rheingold AA Baddeley JL Williams JL Brown C Wallace MM Correa F. Restorative retelling for violent death: an investigation of treatment effectiveness, influencing factors, and durability. J Loss Trauma. (2015) 20:541–55. 10.1080/15325024.2014.957602 26640420 PMC4667545

[54] Rosner R Pfoh G Kotoucova M Hagl M. Efficacy of an outpatient treatment for prolonged grief disorder: a randomized controlled clinical trial. J Affect Disord. (2014) 167:56–63. 10.1016/j.jad.2014.05.035 25082115

[55] Saindon C Rheingold AA Baddeley J Wallace MM Brown C Rynearson EK. Restorative retelling for violent loss: an open clinical trial. Death Stud. (2014) 38:251–8. 10.1080/07481187.2013.783654 24524588 PMC4506689

[56] O'Connor M Piet J Hougaard E The effects of mindfulness-based cognitive therapy on depressive symptoms in elderly bereaved people with loss-related disstress: a controlled study. Mindfulness. (2014) 5:400–9. 10.1007/s12671-013-0194-x

[57] Jacob N Neuner F Maedl A Schaal S Elbert T. Dissemination of psychotherapy for trauma spectrum disorders in postconflict settings: a randomized controlled trial in Rwanda. Psychother Psychosomat. (2014) 83:534–6. 10.1159/000365114 25323203

[58] Schaal S Elbert T Neuner F. Narrative exposure therapy versus interpersonal psychotherapy. Psychother Psychosomat. (2009) 78:298–306. 10.1159/000229768 19628958

[59] Andrade AS Moreira M Sa M Pacheco D Almeida V Rocha JC. Randomized controlled trial of a cognitive narrative crisis intervention for bereavement in primary healthcare. Behav Cogn Psychother. (2017) 45:85–90. 10.1017/S1352465816000345 27618877

[60] van der Houwen K Schut H van den Bout J Stroebe M Stroebe W. The efficacy of a brief internet-based self-help intervention for the bereaved. Behav Res Ther. (2010) 48:359–67. 10.1016/j.brat.2009.12.009 20070953

[61] De Heus A Hengst SMC de La Rie SM Djelantik AAAMJ Boelen PA Smid GE. Day patient treatment for traumatic grief: preliminary evaluation of a one-year treatment programme for patients with multiple and traumatic losses. Eur J Psychotraumatol. (2017) 8:1375335. 10.1080/20008198.2017.1375335 29038679 PMC5632766

[62] Shear MK Reynolds CF III Simon NM Zisook S. Optimizing treatment of complicated grief: a randomized clinical trial. JAMA Psychiatry. (2016) 73:685–94. 10.1001/jamapsychiatry.2016.0892 27276373 PMC5735848

[63] Roberts L Montgomery S. Mindfulness-based intervention for perinatal grief education and reduction among poor women in Chhattisgarh, India: a pilot study. Interdiscip J Best Pract Glob Dev. (2016) 2:1. Available online at: https://knowledge.e.southern.edu/ijbpgd/vol2/iss1/1/28357415 PMC5367631

[64] Roberts L Montgomery S. Mindfulness-based intervention for perinatal grief in rural India: improved mental health at 12 months follow-up. Issues Ment Health Nurs. (2016) 37:942–51. 10.1080/01612840.2016.1236864 27911141

[65] Cupchik GC The evolution of psychical distance as aesthetic concept. Cult Psychol. (2002) 8:155–87. 10.1177/1354067X02008002437

[66] Scheff TJ The distancing of emotion in ritual. Curr Anthropol. (1977) 18:483–90. 10.1086/201928

[67] Morina N Lancee J Arntz A. Imagery rescripting as a clinical intervention for aversive memories: a meta-analysis. J Behav Ther Exp Psychiatry. (2017) 55:6–15. 10.1016/j.jbtep.2016.11.003 27855298

[68] Kalantari M Yule W Dyregrov A Neshatdoost H Ahmadi SJ. Efficacy of writing for recovery on traumatic grief symptoms of Afghani refugee bereaved adolescents: a randomized control trial. OMEGA J Death Dying. (2012) 65:139–50. 10.2190/OM.65.2.d 22953510

[69] Driver T Liberating Rites. Understanding the Transformative Power of Ritual. Charleston, SC: Booksurge (2006).

[70] Turner V The Ritual Process. Structure and Anti-Structure. Piscataway, NJ: Transaction (1969).

[71] Seligman AB Weller RP Puett MJ Simon B. Ritual and its Consequences. Oxford: Oxford University Press (2008). 10.1093/acprof:oso/9780195336009.001.0001

[72] Gersons BPR Meewisse M Nijdam MJ Olff M Protocol Brief Eclectic Psychotherapy for Posttraumatic Stress Disorder (BEPP). Amsterdam: Academic Medical Centre at the University of Amsterdam & Arq Psychotrauma Expert Group (2011).

[73] Doka KJ Therapeutic ritual. In: Neimeyer RA editor. Techniques of Grief Therapy: Creative Practices for Counseling the Bereaved, 1st ed New York, NY: Routledge (2012). p. 341–3.

[74] Crossely N 1 ritual, body technique and (inter)subjectivity. In: Schilbrack K editor. Thinking Through Rituals - Philosophical Perspectives. New York, NY: Routledge (2004).

[75] White C Marin M Fessler DMT Not just dead meat: an evolutionary account of corpse treatment in mortuary rituals. J Cogn Culture. (2017) 17:146–68. 10.1163/15685373-12342196

[76] Norton MI Gino F. Rituals alleviate grieving for loved ones, lovers, and lotteries. J Exp Psychol General. (2014) 143:266–72. 10.1037/a0031772 23398180

[77] Xygalatas D Konvalinka I Roepstorff A Bulbulia J. Quantifying collec- tive effervescence: heart-rate dynamics at a fire-walking ritual. Commun Integr Biol. (2011) 4:735–8. 10.4161/cib.17609 22446541 PMC3306345

[78] Smid GE Drogendijk AN Knipscheer J Boelen PA Kleber RJ. Loss of loved ones or home due to a disaster: effects over time on distress in immigrant ethnic minorities. Transcult Psychiatry. (2018) 55:548–68. 10.1177/1363461518784355 30027823

[79] Hinton DE Good BJ The culturally sensitive assessment of trauma: Eleven analytic perspectives, a typology of errors, and the multiplex models of distress generation. In: Hinton DE Good BJ editors. The Ethnography of Political Violence. Culture and PTSD: Trauma in Global and Historical Perspective. Philadelphia, PA: University of Pennsylvania Press (2016). p. 50–112.

[80] Bhugra D Becker MA. Migration, cultural bereavement and cultural identity. World Psychiatry. (2005) 4:18–24. 16633496 PMC1414713

[81] Doka KJ (editor). Disenfranchised Grief: New Directions, Challenges, and Strategies for Practice. Champaign, Ill Research Press (2002).

[82] Brin D The use of rituals in grieving for a miscarriage or stillbirth. Women Ther. (2004) 27:123–32. 10.1300/J015v27n03_09

[83] Wampold BE Imel ZE The Great Psychotherapy Debate. New York, NY: Routledge (2015). 10.4324/9780203582015

[84] Smid GE Groen S de la Rie SM Kooper S Boelen PA. Toward cultural assessment of grief and grief-related psychopathology. Psychiatr Serv. (2018) 69:1050–2. 10.1176/appi.ps.201700422 30041592

[85] Lewis-Fernández R Aggarwal NK Kirmayer LJ. The cultural formulation interview: progress to date and future directions. Transcult Psychiatry. (2020) 57:487–96. 10.1177/1363461520938273 32838656

[86] Gonçalves OF Machado P Rosas M A elaboração narrativa dos aspectospsico- traumáticos do enfarte miocárdio: um manual terapêutico. Psicolo Teoria Invest Prática. (1997) 3:26–7.

[87] Segal ZV Williams JMG Teasdale JD Mindfulness- Based Cognitive Therapy. New York, NY: Guilford (2002).

[88] Rynerson EK Correa F. Accomodation to Violent Dying. A Guide to Restorative Retelling and Support. (2008). Retrieved from: http://www.vdbs.org/docs/ATVDENGLISH_JUN2013.pdf

[89] Rosner R Bartl H Pfoh G Kotoucova M Hagl M. Efficacy of an integrative CBT for prolonged grief disorder: a long-term follow-up. J Affect Disord. (2015) 183:106–12. 10.1016/j.jad.2015.04.051 26001670

[90] Pennebaker JW Beall SK. Confronting a traumatic event: toward and understanding of inhibition and disease. J Abnorm Psychol. (1986) 96:274–81. 10.1037/0021-843X.95.3.274 3745650

[91] Gonçalves O Psicoterapia cognitiva narrativa: Manual de terapia breve [Cognitive narrative psychotherapy: Handbook of brief therapy]. Bilbao: Editorial Desclee (2002).

