# Supplementary material for: Ritual in Therapy for Prolonged Grief: A Scoping Review of Ritual Elements in Evidence-Informed Grief Interventions
Source: Front Psychiatry. 2021 Feb 3;11:623835. doi: 10.3389/fpsyt.2020.623835 (PMC7887294; doi:10.3389/fpsyt.2020.623835)
Supplement: Supplementary file 1 [file Data_Sheet_1.docx]

**Appendix 1. Evidence-informed interventions for prolonged grief comprising ritual elements**

| Treatment name | Reference | Description | Ritual element(s) | Population^1^ | Study design and data collection | Effect on grief | Level of evidence^2^ |
| --- | --- | --- | --- | --- | --- | --- | --- |
| ATTEND model (mindful-based approach) | Thieleman et al 2014 (46) | Individual sessions, (number and length of sessions not specified) | Various ritual elements, therapist attunes elements:  meditation, (sometimes contemplative prayer), picking symbols/metaphors, therapeutic touch (on top of client’s hand),  funeral/grave visit, take a walk and pick items from nature as metaphors for one’s life | American patients in grief counselling (*n*=42), M age =38.98, *SD*=11.2 | Quasi experiment: Pre/posttest;  Impact of Event Scale-Revised (IES-R)(PTSD) and the 25-item Hopkins Symptom Checklist (HSCL-25) (anxiety & depression) | Symptom reduction on IES-R (p<.001, Cohen’s *d*= .92) and HSCL-25 (p<.001, *d*=.70) | Level 3 |
| Brief eclectic therapy for PTSD/Postraumatic grief (BEPP)  [manual Gersons et al. 2010] (72) | Smid et al. 2018c (50) | 16 individual weekly sessions (each 45-60 min.) | -Mementos: objects that are symbolically linked to the traumatic experience (sometimes used in the farewell ritual)  -Farewell ritual: at end of therapy ritual is conducted with the purpose to leave the traumatic experience behind | Dutch police officers with PTSD (*n*=534), M age=37.6, *SD*=10 | Naturalistic study: Pre/posttest  Police Life Events Schedule (PLES), Trauma Experience Questionnaire (TEQ), Structured Interview for PTSD (SI–PTSD, Clinician‐Administered PTSD Scale (CAPS) | Symptom reduction on PTSD  -treatment effect size (*d*=3.62)  - effect farewell ritual: Coeff. 0.37 (p<.05), 95% CI [−0.75,−0.01], Std. coeff. −.06 (p<.05) 95%CI [−.12, .00 | Level 3 |
| Brief Eclectic Therapy for Traumatic Grief (BEP-TG) [manual Smid et al. 2015] (36) | De Heus et al. 2017 (61) | -16 group sessions (each 75 min.) | -Writing assignments (letter to deceased, angry letter to perpetrator and advice letter imaginary companion)  -Mementos: objects that are symbolically linked to traumatic experience (sometimes used in the farewell ritual)  -Farewell ritual at the end of treatment designed by patient (e.g. burn angry letter, culturally appropriate ritual, renounce traumatic experience), therapist is not present farewell during ritual | Refugees with multiple traumatic losses (*n*=16) from Armenia, Bosnia-Herzegovina, Iraq, Sierra Leone, Syria Hungary, M age =39.7, *SD*=8.8 | -Pre/posttest  -Traumatic Grief Inventory-Self-Report (TGI-SR), Clinician-Administered PTSD Scale (CAPS), | Symptom reduction on CAPS (*d* = 0.93 Intent-to-threat sample, *d=*1.10 completers sample, *n*=13) | Level 3 |
| Cognitive Behavioral Therapy (CBT) with mindfulness | Lenferink et al. 2019 (48) | -8 individual sessions, (each 45 min.)  -4 individual writing exercises (e.g. each day for 20 min or once a week for 2 hours) and 5 times a week mindfulness exercise | -Writing exercise: “write a supportive letter to (hypothetical) friend”  -Mindfulness breathing exercise | Dutch adults with long-term missing loved-one (*n* =17), M age=60.62, *SD* = 13.12 | Pre/posttest (1, 12, 24-week follow-up), Inventory of Complicated Grief (ICG)/ Persistent Complex Bereavement disorder (PCBD), Major Depressive Disorder (MDD), PTSD checklist for DSM-5, Inventory of Depressive Symptomatology Self-Report (IDS-SR), Southampton Mindfulness Questionnaire (SMQ) | No test due to small sample, but symptom reduction on PTSD (Hedges’ *g* from 0.71-0.87), PCBS (Hedges’ g from 0.35-0.57), MDD Hedges’ g from Hedges’ g from 0.97-1.09) | Level 4 |
| Cognitive Narrative Psychotherapy [shorter version of Gonçalves et al 1997] (86) | Andrade et al. 2017 (59) | 4 individual weekly sessions (each 1 hour) | Metaphorization: Finding a metaphor for loss (“if it was a movie or book, what title would you give?” + imagine what deceased would say (“if your … was here, what would you think he would say?” | Portuguese bereaved (*n*= 29) | RCT: control group  Depressive symptoms (BDI), posttraumatic symptoms (IES-R) | Symptom reduction on IES-R Emotional numbing (p<.05, Hedges’ *g*=.80) | Level 2 |
| Cognitive Narrative Psychotherapy [based on Gonçalves 2002] (91) | Barbosa et al. 2014 (52) | 4 individual weekly sessions (each 1 hour) | Metaphorization Finding a metaphor for loss (“if it was a movie or book, what title would you give?” + imagine what deceased would say (“if your …was here, what would you think he would say?” | Portuguese bereaved elders, spousal loss (*n*=40) | RCT: control group  Complicated grief (ICG), Depressive symptoms (BDI), Impact of events scale (IES-R), | Symptom reduction on ICG (p<.01, Hedges’ *g*=2.41), BDI (p<.01, Hedges’ *g*=1.57) & IES-R (p<.01, Hedges’ *g*=3.59) | Level 2 |
| Complicated Grief Treatment (+placebo + antidepressant/citalopram) | Shear et al. 2016 (62) | 16 individual weekly sessions (each 45-60 min.) | Dialogue: Imaginal conversation with the deceased | American bereaved with complicated grief (n = 395), Recruitment 18-95 years | RCT, control group, Grief related symptoms (ICG), Grief Related Avoidance Questionnaire (GRAQ), Work and social impairment (WSAS), Depression (QIDS-SR), Suicidal Ideation (Columbia Suicide Scale) | Effect of CGT on symptom reduction in both antidepressant condition ICG (p<.001, Mean reduction -7,37 (SD 2.08) and GRAQ (p<.001, Mean reduction -5.19 (SD 1.60) and placebo condition ICG (p<.001, Mean reduction -8.01 (SD 2.04) & GRAQ (p<.001, Mean reduction -5.74 (1.43) | Level 2 |
| Integrative Cognitive Behavioral Therapy (PG-CBT) [manual see Rosner et al. 2011] (34) | Rosner et al. 2014 (54) | 20 individual sessions (each 50 min, 2 sessions are 90 mins), 5 optional sessions after treatment | -walk to the grave  -dialogue with deceased (‘what I always still wanted to tell you/ask you’, ‘this is how your death has impacted my life’)  -ritual at the end of treatment  -optional: how to ritualize anniversary, birthday | German bereaved adults with prolonged grief (*n*=51), M age =47.53, *SD*=14.72 | RCT pre/posttest, control group  -German version of Prolonged Grief-13, Psychological distress and mental health checklist Symptom Checklist-90-Revised (SCL-90-R) | Symptom reduction between-group completer analyses: PG-13 (p<.001, η^2^=.47, *d*=1.61), SCL-90-R: depression (p<.01, η^2^=.16, *d*=.60) | Level 2 |
| Integrative Cognitive Behavioral Therapy (PG-CBT) [manual see Rosner et al. 2011] (34) | Rosner et al. 2015 (89) | 20 individual sessions (each 50 min, 2 sessions are 90 mins), 5 optional sessions after treatment | -walk to the grave  -dialogue with deceased (‘what I always still wanted to tell you/ask you’, ‘this is how your death has impacted my life’)  -ritual at the end of treatment  -optional: how to ritualize anniversary, birthday | German bereaved adults with prolonged grief (*n*=41), M age in follow-up sample was ten years younger | RCT 1.5-year follow up (pre/posttest)  -German version of Prolonged Grief-13, Psychological distress and mental health checklist Symptom Checklist-90-Revised (SCL-90-R) | -Symptom reduction completer analyses pre to 1.5-year follow-up:  PG-13 Within group (d=2.22,p<.001), SCL-90-R: global; severity index (*d*=.71, p<.001), depression (*d*=.81, p<.001), somatization (*d*=.56, p<.001), anxiety (*d*=.56, p<.001)  - Symptom reduction intent-to-treat analyses pre to 1.5-year follow-up:  PG-13 Within group (*d*=1.24, p<.001), SCL-90-R: global; severity index (*d*=.51, p<.001), depression (*d*=.54, p<.001), somatization (*d*=.33, p<.05), anxiety (*d*=.38, p<.05) | Level 2 |
| Mindful-based Cognitive Therapy (MBCT) [based on Segal et al. 2002] (87) | O’Connor et al 2014 (56) | 8 group sessions, weekly (each 2-2,5 hours) | Sitting meditation, body scan, breathing and yoga exercises | Danish bereaved elderly (*n*=48), M age=77.7, *SD*=4.5 | Non-randomized, controlled pilot design: Pre/posttest (5-month follow-up)  Beck Depression Inventory (BDI II), Harvard Trauma Questionnaire—Part IV, The Inventory of Complicated Grief—Revised (ICG-R), Centrality of Event Scale (CES), Letter–number sequencing | Symptom reduction on depression (p=.02, Hedges’ *g* =.88), follow-up depression (p=.03)  Improved working memory (p=.044, Hedges’ *g*=.50) | Level 3 |
| Mindfulness-based Stress Reduction (MBSR) | Roberts & Montgomery 2016a (63) | 5 weekly group sessions  (each session about 2,5 – 3,5 hours) | Culturally adapted short version of MBSR:  meditation exercises, gratitude reminders | Indian women with perinatal loss  (*n*= 23), (Age between 18-35) | Pre/posttest (6, 12-week follow-up), Anxiety + depression: Hopkins Symptoms Check List –10 (HSCL-10), Satisfaction With Life Scale (SWLS),  Short form of the Brief RCOPE,  Perinatal Grief Scale (PGS),  Social Provisions Scale (SPS),  Five Facet Questionnaire: Short Form (FFMQ-SF),  Mindfulness exercise practice log,  Program evaluation,  Follow-up evaluation | Symptom reduction on perinatal grief (PGS) (p=<.01), and HSCL-10 (p<.05) | Level 3 |
| Mindfulness-based Stress Reduction (MBSR) | Roberts & Montgomery 2016b (64)  (6-week + 12-month follow up) | 5 weekly group sessions, (each session about 2,5 – 3,5 hours) | Culturally adapted short version of MBSR: meditation exercises, gratitude reminders | Indian women with perinatal  (*n*=22), Age between 18-35 years | Pre/posttest (6 weeks, 12-month follow-up) Anxiety + depression: Hopkins Symptoms Check List –10 (HSCL-10), Satisfaction With Life Scale (SWLS),  Short form of the Brief RCOPE,  Perinatal Grief Scale (PGS),  Social Provisions Scale (SPS),  Five Facet Questionnaire: Short Form (FFMQ-SF), Freiburg Mindfulness Inventory (FMI)  True Resilience Scale (TRS) | Symptom reduction on perinatal grief (PGS) (p=.001), and HSCL-10 (p=.001) | Level 3 |
| Mustard-seed project  (2-day workshop) | Neimeyer & Young-Eisendrath 2015 (44) | 2-day group intervention  (first evening 3 hours, second day 6-7 hours*)* | Various ritual elements: reading of traditional Buddhist story, virtual dreaming (write story with symbols, archetype figures), silent meditation, Dharma lesson (Buddhist spiritual laws), 2 min of silence in a circle at the beginning and the end  - Clinical Psych. + Zen Monk: Stand in circle, silence, mustard-seed story, virtual dreaming, poem, finish “a door closes”, …, end of 2^nd^ day: stand in silence | American adults, Voluntary (paying) participants  (*n*=41/ 35 had lost a loved one/ 6 other unwelcome loss), M age=55 | Open trial: Pre/posttest;  Hogan Grief Reactions Checklist (HGRC) (despair, panic behaviour, blame & anger, detachment, disorganization, personal growth), Integration of Stressful Life Experiences Scale (ISLES) (meaning making of stressful life-events) | Symptom reduction on despair (p=.05) and panic behaviour *(*p=.03)  Improved personal growth (p=.04) & meaning integration (p=.02) | Level 3 |
| Narrative exposure therapy (NET) [based on Schauer et al. 2011] (38) | Jacob et al 2014 (57) | 8 individual weekly sessions (each 90 min-150 min) | Lifeline:  Lay out lifeline with a string, stones (traumas), flowers (positive events), candles and sticks | Rwandan widows (*n* =43) & orphans (*n*=33) after genocide, M age=48.29, *SD*=13.4 | RCT: Pre/posttest (6, 12-month follow-up) Clinician- Administered PTSD Scale (CAPS), 3-month, 6 month, 12-month follow-up | Improvement on CAPS-scale group 1 after treatment (12-month follow up p<.001, Cohen’s *d* = 1.47) and group 2 after treatment (12- month follow-up p<.001, Cohen’s *d*=1.37) | Level 2 |
| Narrative exposure therapy (NET) | Schaal et al. 2009 (58) | 4 individual weekly sessions (each 2-2,5 hours) | Lifeline:  Lay out lifeline with stones (traumas) and flowers (positive events), candles and sticks | Rwandan orphans after genocide (*n*=26), M age=19.42, *SD*=3.59 | RCT (NET/IPT): Pre/posttest (3, 6-month follow-up) Clinician-Administered PTSD Scale (CAPS), Mini-International Neuropsychiatric Interview (MINI), Hamilton Depression Rating Scale, Trauma related guilt + survivor guilt items (secondary outcome) | Symptom reduction on CAPS (p<.01, β=-.28,28), MINI depression score (p<.01, β=-1,83), Hamilton score (p<.05, β=-7,57) | Level 2 |
| Restorative retelling,  [manual by Rynerson & Correa, 2008] (88) | Saindon et al. 2014 (55) | 10 weekly group sessions (each 2 hours) | Various ritual elements: Re-enactment of dying scene (“imagine what loved one heard, saw, felt”), commemoration (client uses photo’s, objects, videotapes to present deceased loved one to group, eat favourite food, listen to specific music) | American bereaved by violent loss (*n*=51), M age=44.84, *SD*=13.35 | Open trial: Pre/posttest: Beck Depression Inventory-A (BDI), Impact of Events Scale, Inventory of Traumatic Grief (ITG), demographics & loss characteristics | -Symptom reduction on depression (p<.005, η^2^=.23), intrusion (p<.005, η^2^=.22), and traumatic grief (p<.005, η^2^=.35), | Level 3 |
| Restorative retelling | Rheingold et al, 2015 (53) [found in systematic review Alves-Costa et al 2019] (41) | 10 weekly group sessions (each 2 hours) | Various ritual elements: Re-enactment of dying scene (“imagine what loved one heard, saw, felt”), commemoration (client uses photo’s, objects, videotapes to present deceased loved one to group, eat favourite food, listen to specific | American bereaved by homicide (*n*=89),  M age=45.34, *SD*=12.71 | Pre/posttest, Uncontrolled Trial,  Impact of Events Scale, Inventory of Traumatic Grief (ITG), Beck Depression, Death Imagery Scale (DI), a.o. | Symptom reduction on PTSD (p<.001, *d*=.46), intrusions (p<.001, *d*=.44), avoidance (p=.001, *d*=.42), hyperarousal (p<.001,*d*=.42), DI (p<.001, *d*=.31), Intervention outcome at 11-month follow-up (n =11), Sustained improvements: PTSD(p,.001, *d*=1.21), CG (p<.001,*d*=1.21), depression (*p*=.006, *d*=.97), no statistically main effect of time: CG | Level 3 |
| Single-Session Music Therapy | Wlodarczyk, 2010 (47) (Dissertation) | Single group session (1 hour) | Group ceremony at end of session with ritual elements: table arrangement, lighting candle, background music, write message to patients that have died, group drumming, put written message in wicker basket, sign initials on dedication certificate, story is read, moment of silence, ringing of chime to end ritual | American hospice employees  (*n*=68) doctors, nurses, social workers, home health aides, clinical support specialists, chaplains, music therapists, M age=46.5 | RCT: Pre/posttest, control group  Hospice Clinician Grief Inventory (HCGI), Marwit-Meuser Caregiver Grief Inventory (MM- CGI), Compassion Satisfaction and Fatigue Test (CSF), Work Environment Scale (WES) , Evaluation “Do you feel that this group has been helpful in resolving your grief feelings from previous patient deaths?” and “Would you like to see a similar group offered regularly at BBH?” Participants circled “yes” or “no” to these questions. | No symptom reduction on HCGI, No significant difference between groups, Positive outcome on personal sacrifice for experiment group (p=0.02) | Level 4 |
| TOZI Healing Retreat | Tuck et al. 2012 (49) [found in systematic review Alves-Costa et al. 2019] (41) | Group retreat over 28 hours span with overnight housing (each session about 45-90 min) | Closing ritual: healing drumming session and commemoration for deceased loved one | American bereaved by homicide (*n*=8), M age=52.2, *SD*=8.8 | Open trial: Pre-posttest (6, 12, 30-week follow-up) General Well-Being Scale (GWB), Center for Epidemiological Studies-Depression-Scale (CES-D), Spiritual Well-Being Scale (SWB), Herth Hope Index (HHI), Revised Impact of Event Scale (IES), PTSD-checklist (PCL-C), Trait Forgiveness Scale (TFS), Transgression-Related Interpersonal Motivations Inventory (TRIM-A, TRIM-B), Single Item Forgiveness Scale (SIF), Texas Revised Inventory of Grief (TRIG), Religious Coping Scale -Brief (RCOPE), | Sample too small for significance tests, TRIG reduction after 6 weeks, but increase after 12 weeks and 30 months | Level 4 |
| Traumatic grief treatment  [based on Shear et al. 2005] (33) | Asukai et al. 2011 (51) | 15 individual weekly sessions (each 1,5 hours*)* | Dialogue:  Imaginary conversation with the deceased (patient is invited to talk with deceased loved one and then respond as the deceased) | Japanese bereaved mothers by violent death (*n*=15), M age=45.15, *SD*=9.81 | Pre/posttest (3, 6, 12-month follow-up), Clinician‐Administered PTSD Scale for DSM‐IV, Inventory of Complicated Grief (CG), Impact of Event Scale‐Revised (IES‐R), Center for Epidemiologic Studies Depression Scale (CES‐D) | Symptom reduction CG (p<.001, *d*=1.72), intrusion (*p*<.001, *d*=1.97) avoidance (p<.001, *d*=1.87)) hyperarousal (p<.001, *d*=1.62), depression (p<.01, *d*=0.99) | Level 3 |
| Writing assignments [based on Pennebaker & Beall, 1986] (90) | Van der Houwen et al. 2010 (60) | 5 individual writing assignments (each 15 min.) | Letter: Writing assignment letter of advice to imaginary friend | Dutch bereaved adults (*n*=757), M age=43.33, *SD*=10.98 | Random assign intervention + control group, grief reactions, depression, positive mood, emotional loneliness  Complicated grief measured by 9 items based on DSM-V criteria, Depression (CES-D), Positive mood (PANAS), Emotional loneliness (2 items) | Positive effect on positive mood (p<.05, *B*=.071), reduction emotional loneliness (p<.05, *B*=-.28) | Level 2 |
| Writing for Recovery | Kalantari et al. 2012 (68) | 3-day group session (each 15 min.) | -Writing for 15 min. for 3 consecutive days and put writing in a blue box after each session  -Final session: “imagine that 10 years have passed: what have you learnt from that experience?” | Afghan bereaved, refugee children (*n*=61), M age =14.58, *SD*=1.68 | -Pre/post-test, control group  -Traumatic Grief Inventory for Children (Farsi) (TGIC) | Symptom reduction on traumatic grief TGIC (p<.001, partial eta squared 0.19) | Level 3 |

^1^ If in the study the reported mean age was presented for experimental and control group separately, the here reported mean age refers to the experimental/treatment group. If the mean age was not reported, the age range is noted here.

^2^ Level of evidence according to Oxford Center for Evidence-based Medicine 2011, OCEBM Levels of Evidence Working Group. Level 1 Systematic review of randomized trials, Level 2 Randomized trial or observational study with dramatic effect, Level 3 Non-randomized controlled cohort/follow-up study, Level 4 Case-series, case control, or historically controlled studies, Level 5 Mechanism based reasoning.
